# Interferometric Dynamic Measurement: Techniques Based on High-Speed Imaging or a Single Photodetector

**DOI:** 10.1155/2014/232906

**Published:** 2014-05-12

**Authors:** Yu Fu, Giancarlo Pedrini, Xide Li

**Affiliations:** ^1^Temasek Laboratories & School of Mechanical & Aerospace Engineering, Nanyang Technological University, 50 Nanyang Drive, Singapore 637553; ^2^Institut für Technische Optik, Universität Stuttgart, Pfaffenwaldring 9, 70569 Stuttgart, Germany; ^3^Department of Engineering Mechanics, AML, CNMM, Tsinghua University, Beijing 100084, China

## Abstract

In recent years, optical interferometry-based techniques have been widely used to perform noncontact measurement of dynamic deformation in different industrial areas. In these applications, various physical quantities need to be measured in any instant and the Nyquist sampling theorem has to be satisfied along the time axis on each measurement point. Two types of techniques were developed for such measurements: one is based on high-speed cameras and the other uses a single photodetector. The limitation of the measurement range along the time axis in camera-based technology is mainly due to the low capturing rate, while the photodetector-based technology can only do the measurement on a single point. In this paper, several aspects of these two technologies are discussed. For the camera-based interferometry, the discussion includes the introduction of the carrier, the processing of the recorded images, the phase extraction algorithms in various domains, and how to increase the temporal measurement range by using multiwavelength techniques. For the detector-based interferometry, the discussion mainly focuses on the single-point and multipoint laser Doppler vibrometers and their applications for measurement under extreme conditions. The results show the effort done by researchers for the improvement of the measurement capabilities using interferometry-based techniques to cover the requirements needed for the industrial applications.

## 1. Introduction

In the last several decades, the drive for higher performance and reliability of devices, structures, and processes in engineering has placed stringent demands on the methods used in their development and measurement. Optical metrology is a major and indispensable part of the measurement methods. The field of optical metrology is arguably more than one century old. However, major advances have resulted from the invention of laser only about fifty years ago. This new light source opened a realm of new techniques to both the physicist and the engineer. With the advent of the laser, coherent optics has been brought into measurement techniques of various areas. Optical interferometry [1] is a well-known technique to measure the path-length change of laser light caused by deformation, movement, or unevenness of an object. Generally, there are two types of techniques: one is based on two-dimensional sensor like CCD or CMOS camera and the other is based on single-pixel photodetector.

### 1.1. Interferometric Dynamic Measurement (IDM)

The typical camera-based interferometry includes different methods, such as photoelasticity [2], moiré interferometry [3], electronic speckle pattern interferometry (ESPI) [4], shearography [5], digital holography [6], and white-light interferometry [7]. Generally, they are noncontacting and whole-field techniques providing results in the form of fringe patterns that represent different physical quantities, such as distance, in-plane or out-of-plane displacements, strain, stresses, or refractive-index. Although a fringe pattern representing distance, deformation, or distortion is readily obtained, expert interpretation is necessary to convert these fringes into the desired information. For the accurate mapping of these physical quantities various fringe processing algorithms, notably the Fourier transform [8] and phase shifting [9], have been used. Due to the limited capturing rate of the camera and the long processing time for fringe pattern, initially these interferometers were mainly used for quasi static measurements. For high-frequency periodical vibration measurement, the camera-based interferometry is applied to determine vibration modes of objects. Time-average methods, based on digital holography [10], ESPI [11], DSSI [12], or moiré [13], directly acquire a spatially dense, full-field, real-time image of the mode shape, while other techniques require the reconstruction of the mode shape from single point measurements. Methods using stroboscopic light sources [14] allow “fixing” the steady-state vibration and can be applied in principle for the measurement of nonsinusoidal vibrations, but the movement should be periodical. In addition, if low-speed CCDs are used, the time for acquiring the interferograms may be quite long and this makes the technique poorly suited to be used in an industrial environment.

In many cases, high-resolution three-dimensional (3D) dynamic displacement or surface profiling can give useful information about the dynamic response of a mechanical structure. However, it is difficult to achieve this with the abovementioned techniques. The use of double-pulse laser interferometry [15] has been reported as an alternative to obtain transient deformations. However, this technique has a fatal limitation, since, to obtain the evolution of the transient deformation, an experiment must be repeated many times with a different interval of two pulses. This means that nonrepeatable events cannot be studied in detail. Due to the rapid development of high-speed digital recording devices, it is possible to record interferograms with rates exceeding 100,000 frame/s. In order to measure various physical quantities at any instant, high-speed camera is adopted in various interferometric techniques to satisfy the Nyquist sampling theorem along time axis. This leads to a series of interferometric dynamic measurement techniques based on high-speed imaging.

On the other hand, single-pixel detector-based interferometry has already been successfully applied for dynamic measurement. Laser Doppler vibrometry [16] and laser Doppler velocimetry [17] are two similar techniques. The former is for the measurement of out-of-plane displacement or velocity and the latter is for in-plane displacement or velocity measurement. Compared to a full-field measurement based on high-speed camera, detector based interferometry can only offer a point-wise measurement, but with large measurement velocity range. For the extraction of the Doppler frequency shift, different interferometric solutions can be used. The heterodyne Mach-Zehnder and the homodyne Michelson interferometers are two typical configurations. In order to measure the vibration at different points, laser Doppler vibrometers are equipped with a video camera and a scanning system. These scanning laser Doppler vibrometers (SLDV) give the possibility of moving the measurement point rapidly and precisely on the testing surface, allowing the analysis of large surface with high spatial resolution [18].

### 1.2. Current Status of IDM

**Figure 1:**
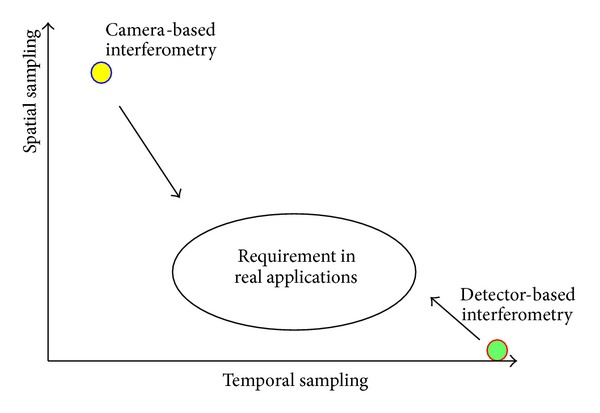
The current status of camera-based and detector-based interferometry.

Figure 1 shows that the camera-based technique has very high spatial resolution but its temporal resolution is low compared with the requirement of real applications. Even a 100 k frame/s capturing rate sometimes cannot satisfy the Nyquist sampling theorem; as for every measurement point, the phase change between two consecutive images should be less than *π*. In addition, a high power laser is needed for the illumination and the cost of high-speed camera also limits the applications in industrial areas. On the contrary, detector-based laser Doppler techniques have enough sampling rate along the time axis, but only one measurement point in spatial domain. A scanning device is usually used to perform 2D measurements. This method assumes that the measurement conditions remain invariant while sequential measurements are performed. Hence, it is only suitable to measure steady-state or well-characterized vibrations. However, most engineering applications do not satisfy these requirements. Transients, including impact or coupled vibrations, are commonly observed in real applications and scanning LDVs impractical to generate a vibration image in these cases.

In this paper, some new measurement and processing technologies based on high-speed imaging and single detector are reviewed. Several issues of the camera-based IDM are discussed; these include the carrier and processing domain, phase extraction technique, and how to increase the measurement range by using the multiwavelength method. A detector-based IDM technique using a multipoint laser Doppler vibrometer (LDV) based on a spatially-encoded technology is described. Some applications under extreme conditions are presented and the results show the trend of IDM to meet the requirements necessary for industrial applications with sufficient temporal and spatial sampling points.

## 2. High-Speed-Imaging-Based IDM

**Figure 2:**
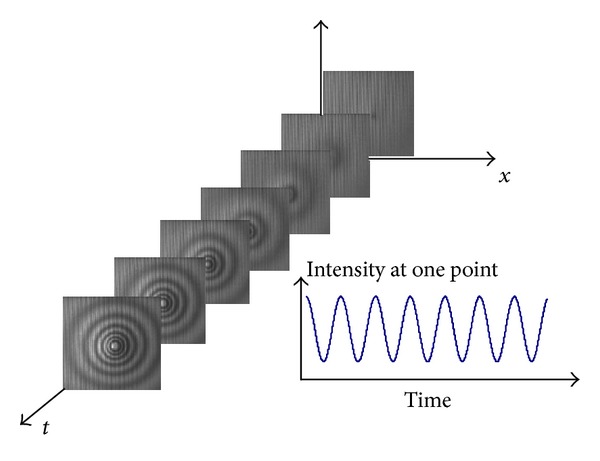
Schematic layout of the high-speed-imaging-based IDM.

In the high-speed-imaging-based interferometric dynamic measurement technique (IDM), the signal obtained is a sequence of interferograms recorded at time *t* (see Figure 2) that can be written as

(1) f(x,y;t)=Ib(x,y;t) +A(x,y;t)cos⁡(φ(x,y;t)+φ0(x,y)),

where *I*_*b*_(*x*, *y*; *t*) and *A*(*x*, *y*; *t*) are the intensity bias and the modulation factor, respectively. Both items are slowly time-varying functions. *φ*(*x*, *y*; *t*) is a phase variation due to a vibration or a continuous displacement. *φ*_0_(*x*, *y*) is the initial phase value of each pixel. The purpose of the imaging-based IDM is to extract the phase *φ*(*x*, *y*; *t*) from a 3D matrix as expressed in (1).

### 2.1. Phase Extraction Techniques

There are quite a lot of phase extraction techniques for interferometric fringe patterns and these are classified into three categories: phase-shifting methods, transform-based methods, and others.

#### 2.1.1. Phase-Shifting Techniques

The phase-shifting techniques can be classified into temporal and spatial phase shifting. In the temporal phase shifting technique, at least three phase-shifted fringe patterns or specklegrams are collected. In order to keep the phase nearly unchanged during the phase shifting, a fast phase shifter and a high capturing rate are necessary. In 1996, de Lega and Jacquot [19] proposed the object-induced temporal phase changes where a piezoelectric transducer (PZT) with the driven frequency of 80 Hz was used to produce a phase shift between two consecutive frames. In 1999, Huntley et al. [20] developed a phase shifting speckle interferometer operated at 1 k Hz capturing rate using a high-speed camera. A Pockels cell producing a phase shift of *π*/2 between two consecutive frames was used and the system was synchronized with the camera. In 2003, Kaufmann [21] used a similar system to monitor the out-of-plane deformation of a plate with flaw. In these techniques, temporal phase unwrapping [22] was applied. Hence, the measurement errors were also accumulated. In 2002, Kao et al. [23] applied the phase shifts to the initial status *t*_0_ but not to other instances *t*. Phase-shifted speckle-correlation fringe patterns can be formed between *t*_0_ and *t*. This method is very simple but suffers from speckle decorrelation. In 2005, the rereferencing rate, that is, the update rate of the reference frame, was studied [24] to avoid the influence of the speckle decorrelation. Generally, the temporal phase-shifting method is only suitable to measure a slow-varying deformation or profile changing producing a phase variation which is much slower compared with the temporal carrier generating the reference phase shift.

In the spatial phase-shifting techniques, several phase-shifted fringe patterns are captured in one shot at different locations by either different cameras or on different areas of a camera. Several such systems are described in [25–27]. A significant innovation is a camera with a pixelated phase mask that can be combined with different interferometers [28, 29]. This technique converts a  2 × 2  superpixel into four phase-shifted pixels using micropolarizers, avoiding the registration of several phase-shifting fringe patterns. Compared to temporal phase-shifting techniques, the measurement range along the time-axis is increased, with the cost of slightly sacrificing the spatial resolution. Similar technique can be found in fringe projection profilometry [30], although this is not considered as an interferometric technique.

#### 2.1.2. Transform-Based Techniques

Transform-based techniques are the predominant phase extraction methods in the imaging-based IDM. These techniques include Fourier transform [8], Hilbert transform [31], windowed Fourier transform [32], wavelet transform [33], and a combination of Fourier and windowed Fourier transform [34]. Like the phase-shifting techniques, the transform-based phase extraction methods can be applied in the spatial domain, temporal domain, or even spatial-temporal domain. The processing can be one-dimensional (1D), 2D, or even 3D. The only requirement is that a carrier has to be introduced along one-axis in the processing domain to avoid the phase ambiguity. Carrier-based 2D spatial Fourier transform [8] was firstly applied in IDM as it allows extracting the phase distribution from a single carrier fringe pattern. It has been applied to measure the transient phenomena by moiré interferometry [35] and speckle interferometry. A spatial carrier leads to a fringe pattern with various fringe densities in one image. However, in the speckle interferogram, the fringe density cannot be too high due to the speckle noise and this limits the measurement. In recent years, other transforms such as windowed Fourier transform [32] and wavelet transform [33] have also been used to process the carrier fringe pattern.

After the introduction of the high-speed camera in IDM, a temporal version of the Fourier analysis and other transform-based methods were applied to extract the phase. In this case, a temporal carrier is required. In 1998, Joenathan et al. performed a series of studies on temporal phase evaluation through speckle interferometry for out-of-plane deformation [36], in-plane deformation [37], the derivative of out-of-plane deformation [38], and the shape measurement [39]. The influence of decorrelation, speckle size, and nonlinearity of the camera were also discussed [40] and a rotating half-wave plate was proposed to introduce a temporal carrier. In 2002, Kaufmann and Galizzi compared the temporal phase-shifting method with the temporal Fourier transform method [41]. In 1997, de Lega firstly described a temporal wavelet phase extraction algorithm for dynamic measurement [42]. The research was continued by Fu et al. [43, 44] as well as Federico and Kaufmann [45]. The S transform, a similar algorithm to wavelet transform, has also been used for temporal phase evaluation [46]. Another technique is the windowed Fourier transform (WFT). In 2003 Ruiz et al. elegantly linked a temporal phase-shifting algorithm to the temporal windowed Fourier transform and showed that the WFT provided better performance [47]. In 2006, Qian et al. applied a 3D WFT to process a sequence of fringe pattern from speckle interferometry [48]. In 2007, Fu et al. applied the WFT to digital holography for vibration measurement and demonstrated its superior performance over the Fourier transform [49]. In the same year, they proposed a combination of two transforms, namely, the temporal Fourier transform and the spatial windowed Fourier transform, and showed that this combination performed better than either single transform [34].

#### 2.1.3. Miscellaneous Algorithms

Another simple but effective temporal phase extraction algorithm, for time sequence analysis, was proposed by Li et al. in 2001–2004 [50–52]. It includes phase scanning method, sequence pulse counting method, and matched correlation sequence analysis. These methods retrieve the phase from the variation of the gray values. The significant advantage of the time sequence analysis algorithm is that it is simple and efficient. The drawback is that it cannot be applied to a very noisy signal as the accuracy of the methods relies on the correct identification of the fluctuations of the interference intensity in each cycle. Li et al. also applied this algorithm to speckle fringe patterns but the method is more suitable for low noise patterns obtained by incoherent methods such as fringe projection and shadow moiré. Furthermore, it needs enough sampling points in one cycle of gray value variation. Li et al. mentioned at least 6 sampling points, but from the experiments it was found that 10 to 16 sampling points per cycle produce the best results.

In recent years, some algorithms for the extraction phase values from one fringe pattern without spatial carrier were reported [53–57]. However, these algorithms are based on some assumptions and they may be working on some fringe patterns but fail on others. Hence, adding a carrier frequency is still the most reliable and effective method.

### 2.2. Carrier and Processing Domain

**Figure 3:**
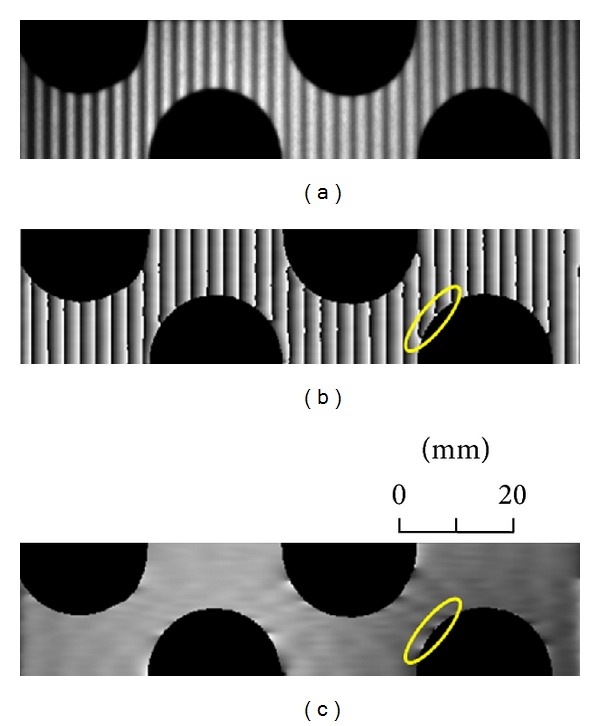
(a) Typical fringe pattern on a cantilever beam with irregular shape; (b) wrapped phase obtained by 2D spatial transform; (c) phase distribution after unwrapping and removal of the carrier.

For the interferometric dynamic measurement, the carrier can be introduced either in the spatial domain (along *x*- or *y*-axis) or in the temporal domain (along the *t*-axis).  Table 1 shows the comparison of spatial and temporal carrier-based techniques. In the spatial-carrier-based technique, the quality of the retrieved phase map is affected by several factors, such as speckle noise, nonuniform reflectivity, and irregular shape of the surface.  Figure 3 gives an example of the effect of irregular shape of surface in the fringe projection technique. Although fringe projection is not an interferometric method, the problem involved in the fringe processing is the same as in interferometry. In fringe projection the carrier already exists in the spatial domain. However, the result of the spatial processing will be seriously affected by a nonuniform reflectivity of the surface or a height step on the surface. Zero-padding is also needed for an irregular shape and this will also generate large error at the edge.  Figure 3(a) shows a fringe pattern projected on a cantilever beam with irregular shape. Figures 3(b) and 3(c) show a wrapped phase map after 2D Fourier transform and the phase distribution after unwrapping and removal of the carrier. The phase distortion close to the edge is due to the zero-padding. The results obtained by WFT and CWT are worse due to their lower resolutions in the spatial and frequency domains. This example shows that spatial processing may not be a good choice in some cases.

**Figure 4:**
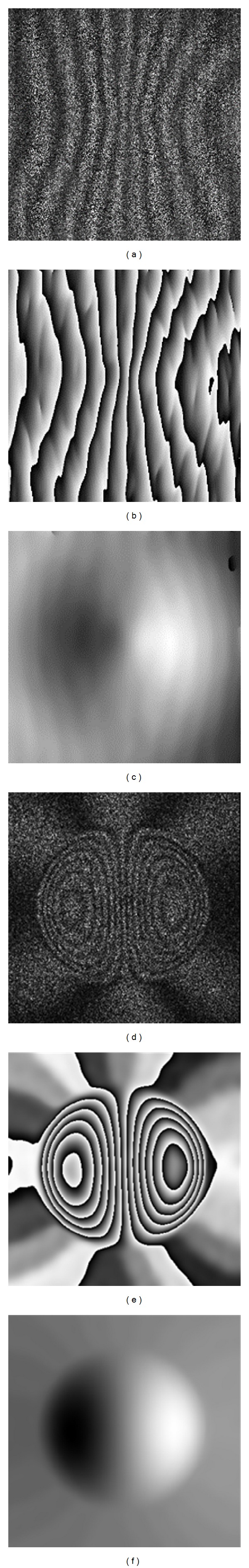
(a) Typical shearography fringe pattern, (b) wrapped phase map, and (c) phase after unwrapping and removal of the spatial carrier; (d) shearography fringe pattern, (e) wrapped phase map, and (f) phase after unwrapping when the temporal carrier is applied (reprint from [58]).

Figure 4 shows a comparison of spatial and temporal carriers in digital shearography [58]. In this technique, the phase change represents the deflection derivative.  Figure 4(a) shows a shearographic fringe pattern with spatial carrier on a fully clamped circular plate with central-point loading where the nearly straight parallel carrier modulates the shearography fringes. The density of the carrier fringes should be high enough to enable the unambiguous determination of the fringe orders. A two-dimensional Fourier transform is then applied to extract the phase from one fringe pattern. The quality of the phase map depends on the proper selection of the band-pass filtering window.  Figure 4(b) shows the best result obtained. However, the noise effect is still obvious on the phase map.  Figure 4(c) shows the unwrapped phase map after removal of the carrier.  Figure 4(d) shows a typical shearography fringe pattern obtained from a dynamic measurement when a temporal carrier is introduced. The fringe density is much higher in this case. The wrapped phase map after temporal analysis and unwrapping are shown in Figures 4(e) and 4(f), respectively. We may see that good results can be obtained even when the fringes are dense.

**Figure 5:**
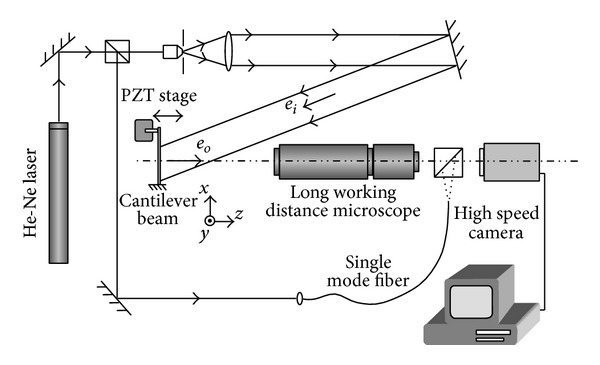
Schematic layout of an image-plane digital holographic microscope (reprint from [59]).

This example shows that the processing along the time-axis usually gives better results. However, due the capturing rate of camera a carrier in the temporal domain will dramatically reduce the measurement range along the time-axis limiting the applications. Hence, a compromise is necessary between these two techniques. This leads to a tradeoff processing in the spatial-temporal domain. The carrier is still in the spatial domain but a 2D algorithm (FFT or WFT) is applied in the spatial-temporal domain [59].  Figure 5 shows an image-plane digital holographic microscopy setup sensitive to out-of-plane displacements. A spatial carrier is introduced by proper positioning of the fiber tip carrying the reference beam. A sequence of digital holograms is captured during the vibration of cantilever beam excited by a PZT.

**Figure 6:**
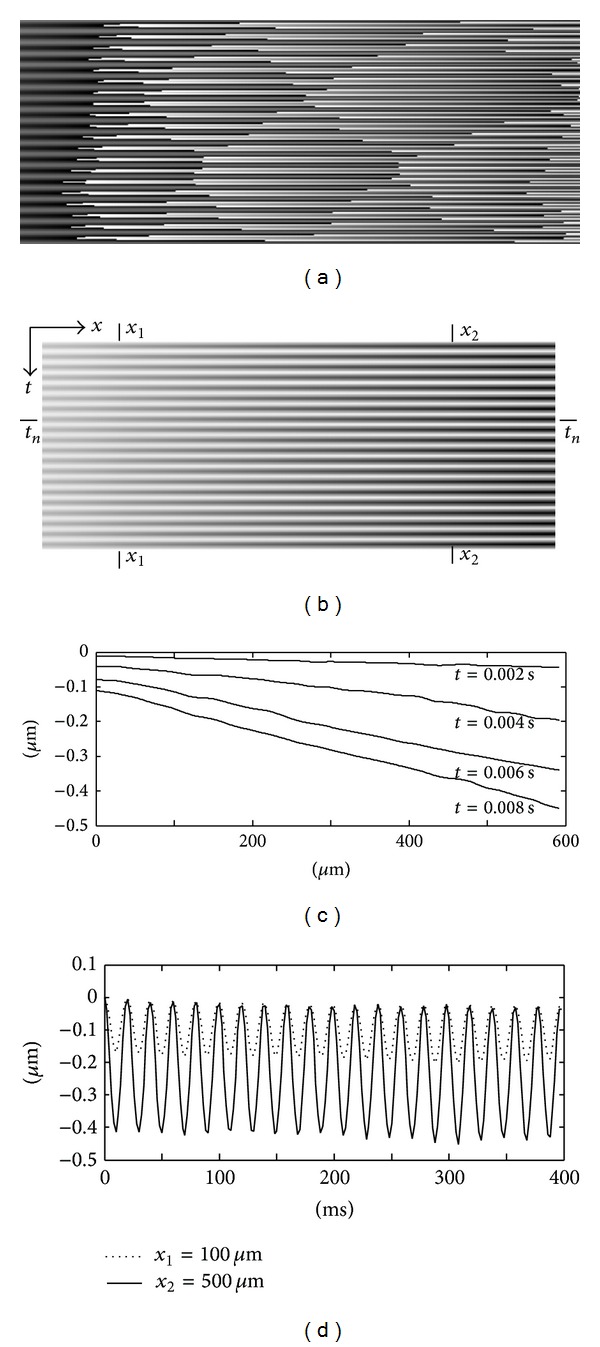
(a) Wrapped phase map in the spatiotemporal plane; (b) unwrapped phase map shows the displacement variation in the spatiotemporal plane; (c) displacement distributions of the cantilever beam at different instants; (d) Displacements of two points on the cantilever beam (reprint from [59]).

A 2D Fourier analysis is applied at each interferogram to obtain a series of 2D wrapped phase maps. The temporal phase variations along the central-line of the cantilever beam generate a spatiotemporal distribution as shown in Figure 6(a). Phase unwrapping along the time axis yields a continuous phase map (Figure 6(b)), from which the instantaneous displacement along the central-line can be calculated.  Figure 6(c) shows the displacement of the beam at different time *t*_*n*_.  Figure 6(d) shows the displacement variation of two points located on the central line at the positions *x*_1_ = 100 *μ*m and *x*_2_ = 500 *μ*m. The phase values in Figure 6(a) are then converted to the exponential values exp⁡(*j* · Δ*ϕ*) and processed in the spatial-temporal domain by using the windowed Fourier ridge algorithm to extract the instantaneous velocity and acceleration.

### 2.3. Dual-Wavelength Technique

In many IDM cases, the frequency of the vibrating object is low and the amplitude is large. As the measurement still needs to satisfy the Nyquist theorem, the requirement on capturing rate should still be very high. In this case, a longer wavelength can effectively increase the measurement range. Lasers emitting infrared radiation (e.g., 10.6 *μ*m CO_2_ laser) are available but unfortunately for such wavelengths the detectors are very expensive and have limited resolution. Hence, dual and multiwavelength techniques were introduced to generate a longer synthetic wavelength. Different types of dual-wavelength interferometers have been reported during the past several decades. A typical application of dual-wavelength is the measurement of a surface profile with height steps [60]. The method can also be used with digital holography for surface profiling where the phase at single wavelength can be reconstructed from separately recorded digital holograms [6, 61]. However, it is not suitable for dynamic measurement since two holograms need to be recorded and reconstructed individually. Here a dual-wavelength interferometer combined with image-plane digital holography is presented to achieve a dynamic measurement on a vibrating object. The object was simultaneously illuminated by two lasers with different wavelengths, and a sequence of digital holograms was captured by a CCD camera [62].

**Figure 7:**
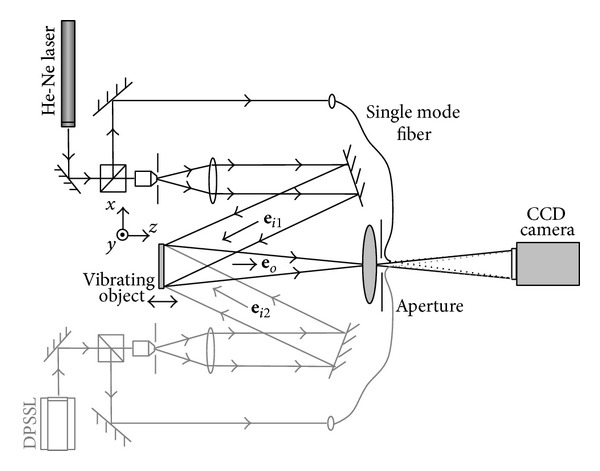
Schematic layout of dual-wavelength image-plane digital holography for dynamic measurement (reprint from [62]).

A schematic layout of a dual-wavelength image-plane digital holography configuration, sensitive to out-of-plane displacement, is shown in Figure 7. Two lasers with different wavelengths are used. Light from the first laser is split into an object beam and a reference beam. This object beam illuminates a vibrating specimen with a diffuse surface along a direction  **e**_*i*1_  . Some light is scattered in the observation direction  **e**_*o*_  where an image-plane hologram is formed on the CCD sensor, as a result of the interference between the reference beam and the object beam. An aperture is put immediately behind the imaging lens to limit the spatial frequencies of the interference pattern. Similarly a second different laser wavelength is used to generate a second interferogram on the CCD sensor. When these two lasers simultaneously illuminate the object and the detector, the two interferograms will be superimposed on the CCD sensor and one digital hologram containing information about these two interferograms will be obtained.

**Figure 8:**
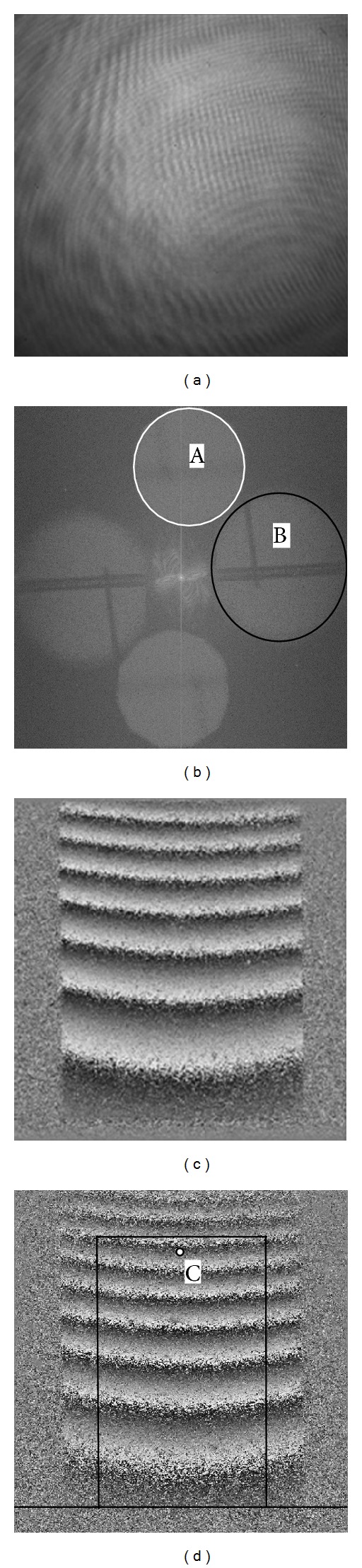
(a) Typical digital hologram obtained by illumination from two lasers; (b) spectrum of the digital hologram obtained; (c) typical original wrapped phase map with *λ*_1_ = 633 nm; (d) typical original wrapped phase map with *λ*_2_ = 523 nm, and the selected area to process (reprint from [62]).

Figure 8(a) shows a typical digital hologram captured by the CCD camera. With a proper selection of the aperture size and a careful adjustment of the two fiber-end positions, it is possible to separate the spectra of two superimposed holograms in the frequency domain. In our experiment, one hundred and twenty holograms are captured during an eight-second period.  Figure 8(b) shows a typical Fourier spectrum of digital holograms captured by the CCD camera. The shadow of the fiber ends can be observed on the spectra of both digital holograms. When* filtering window A* is selected, the reconstructed phase difference between the two instants represents the out-of-plane displacement for the wavelength 632.8 nm (Figure 8(c)). When* filtering window B* is selected, the reconstructed phase between the two instants is the result with wavelength of 532 nm (Figure 8(d)). A slight difference can be observed between these two wrapped phase maps. At each instant *t*_*m*_, a new phase distribution is calculated directly by the subtraction of these two wrapped phases:

(2) $$\begin{array}{c} \Phi =\{\begin{array}{cc} \Delta \phi_{1}-\Delta \phi_{2} & \text{if}\;\;\Delta \phi_{1}\geq \Delta \phi_{2},\\ \Delta \phi_{1}-\Delta \phi_{2}+2\pi & \text{if}\;\;\Delta \phi_{1}<\Delta \phi_{2}, \end{array} \end{array}$$

where 0 ≤ Φ < 2*π*. This phase map is equivalent to a phase distribution of an out-of-plane displacement measurement with a synthetic wavelength Λ, where

(3) $$\begin{array}{c} \Lambda =\frac{\lambda_{\text{eq}1}\lambda_{\text{eq}2}}{\left|\lambda_{\text{eq}1}-\lambda_{\text{eq}2}\right|}. \end{array}$$

**Figure 9:**
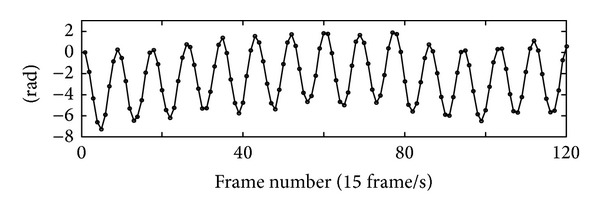
Phase variation of the point C with the synthetic wavelength of 3342 nm which is proportional to the displacement of the object (reprint from [62]).

Figure 9 shows the phase variation of point C (indicated in Figure 8(d)) after 1D temporal phase unwrapping. In our experiment, the synthetic wavelength equals 3342 nm. In this case, the measurement range in the temporal domain has been increased by 5 times. This is a typical technique to increase the temporal measurement range with the cost of sacrificing some resolution in the spatial domain, which shows the efforts to balance the temporal and spatial resolutions in IDM.

## 3. Detector-Based IDM

Detector-based interferometry is usually used for high temporal resolution point-wise measurements and allows measuring large vibrations amplitudes and frequencies. Two specifications of various single-pixel detectors make the detector-based IDM an effective method for industrial applications: (1) large pixel area and (2) wide frequency response. The detectors size is more than several tens of micrometer; many of them are >0.5 mm with an output bandwidth of 1 GHz. Compared to CCD or CMOS cameras, they are more sensitive to low-power laser light. Typical detector-based interferometry includes two similar technologies: laser Doppler vibrometer for out-of-plane displacement or vibration measurement [16] and laser Doppler velocimeter for in-plane velocity measurement [17]. Here we will focus on the laser Doppler vibrometer (LDV) and its applications.

### 3.1. Conventional Single-Point Laser Doppler Vibrometer

**Figure 10:**
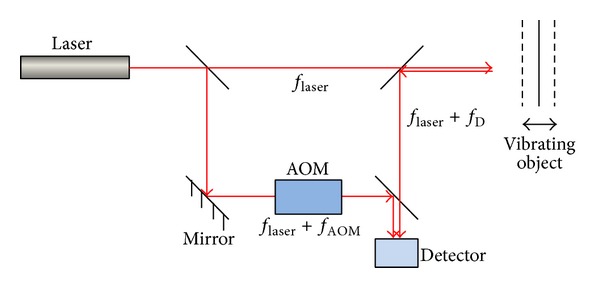
Schematic layout of the heterodyne laser Doppler vibrometer.

The laser Doppler vibrometer is based on the Doppler Effect that occurs when the laser light is scattered from a moving surface. The instantaneous velocity of the surface is converted to the Doppler frequency shift of the laser light which can be extracted by interference between the object and the reference beams. Two configurations have been developed to avoid the directional ambiguity problem: the heterodyne (Mach-Zehnder interferometer including one detector and one acousto-optic modulator) and the homodyne (Michelson interferometer with two detectors and polarization components).  Figure 10 shows the schematic layout of a typical heterodyne single-point laser Doppler vibrometer.

In a single-point LDV, a laser beam with wavelength *λ* is projected on an object moving with velocity  **V**; due to the Doppler effect the shifted frequency *f*_*D*_ of the reflected beam is proportional to the velocity of the object and can be expressed as

(4) $$\begin{array}{c} f_{D}\left(t\right)=\frac{V\left(t\right)\cdot S}{\lambda }, \end{array}$$

where  **S** = **e**_*i*_ − **e**_*o*_  is the sensitivity vector given by the geometry of the setup, and  **e**_*i*_  and  **e**_*o*_  are the unit vectors of the illumination and observation, respectively. In order to avoid the directional ambiguity in frequency shift, the most common solution is the heterodyne interferometer where an optical frequency shift is introduced into one arm of the interferometer by an acousto-optic modulator (AOM) to obtain a virtual velocity offset. The intensity fluctuation at the detector can be expressed as [63]

(5) $$\begin{array}{c} I=I_{DC}+I_{RO}\cos\left(2\pi \left(f_{D}+f_{\text{AOM}}\right)t+\Delta \phi \right), \end{array}$$

where *f*_*D*_ and *f*_AOM_ are the Doppler frequency shift and the carrier frequency introduced by the AOM, respectively. Δ*ϕ* is the phase difference between the reference and object beams. The modulation factor *I*_*RO*_ is the product of the square root of object and reference beam intensities $\sqrt{I_{R}I_{O}}$. The photodetector will convert the intensity fluctuation to a current signal for later analog or digital decoding.

Most of the vibrometric systems offer point-wise measurement. And in order to measure vibrations at different points, a scanning system is used to move the measurement point rapidly and precisely on the testing surface [18]. This technique works only when the measurement conditions remain invariant during the sequential detection. Hence, it is only suitable to measure steady-state or well-characterized vibrations. Unfortunately, most engineering applications do not satisfy these requirements. Transients, including impact or coupled vibrations, are commonly observed in real applications.

### 3.2. Multipoint Laser Doppler Vibrometer

In recent years, several types of multichannel and multipoint LDVs have been reported [64, 65]. This novel idea first appeared in a scientific paper where Zheng et al. [66] proposed a multichannel laser vibrometer based on a commercial single-point Polytec vibrometer and an acousto-optic beam multiplexer. It is still a point-wise measurement but with a switch among different channels instead of a scanning mechanism. Now some robust prototypes [67] and even customer-designed commercial products are available [68]. However, these multipoint versions are usually a combination of several sets of single-point vibrometers systems [69], where multiple detectors or detector array is used [70]. Synchronization is still needed among detectors. Recently some simultaneous multipoint measurements [17, 71, 72] using one laser source and one detector have been reported. These techniques use at least two acousto-optic devices to generate various frequency shifts at spatially-separated points and resolve the signals in frequency domain. However, the measurement results only on two or three points were presented, where cross-talk region can be easily identified in the spectrum. When this approach is applied all object beams will interfere with each other and with a common reference beam. Resolving the measurement signals from cross-talk regions is difficult when the number of measurement points is increasing.

A method based on spatial-encoding [73] was presented recently to overcome the abovementioned problems in the multipoint LDV system and realize a simultaneous vibration measurement on 20 points using one laser source and one single-pixel detector [74]. Twenty laser beams with various frequency shifts were generated by four AOMs at Raman-Nath and Bragg regions. These twenty laser beams were projected onto a vibrating object. The reflected beam array is collected and interferes with a reference beam. The detected interference signal can be expressed by

(6) I=IDC+∑i=120IM(i)cos⁡(2π(fD(i)+fAOM(i))t+Δϕ(i))+∑m=119 ∑n>m20Imncos⁡(2π[(fD(m)−fD(n))+(fAOM(m)−fAOM(n))]t+Δϕmn),

where  *i*,  *m*, and *n* are integers,  *f*_AOM(*i*)_ are the central frequencies of twenty object beams. The second term is the interference signal between twenty object beams and reference beam, from which the useful vibration information of twenty points can be extracted. The third term is the sum of the cross-talk between any two object beams, which has to be avoided when the interference signal is decoded. The carrier frequencies of twenty laser beams have to be elaborately designed so that the useful signals can be separated from the cross-talk regions in frequency spectrum or temporal-frequency spectrogram [75]. Several methods were proposed to bypass the effect of cross-talk. Considering the current capability of A-D converter, a half-step frequency shift was proposed. The disadvantage of this technique is that the velocity measurement range is limited due to the cross-talk of the object beams. However, this will not limit the proposed multipoint LDV in normal engineering applications. This method increases the spatial measurement points with the cost of sacrificing the measurement range in the temporal domain.

### 3.3. Applications of LDV

Two applications are presented in this section to show the capability of single-point and multipoint LDV. One is an application of single-point LDV in a scanning near-field optical microscope (SNOM); and the second is a transient event measurement by a four-point LDV.

#### 3.3.1. Single-Point LDV for Shear-Force Dynamics in the SNOM

**Figure 11:**
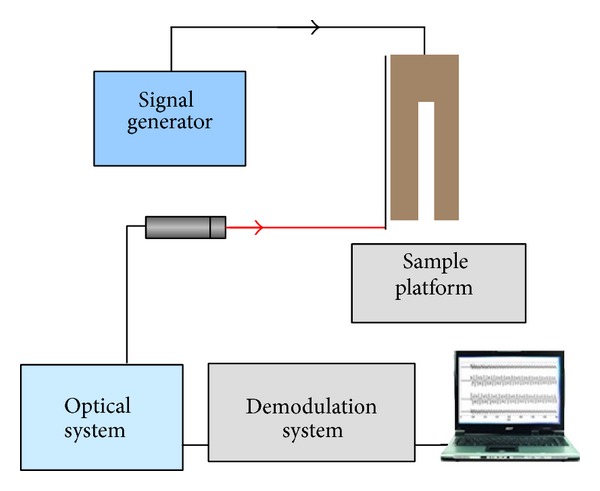
Schematic diagram of single-point LDV based tuning fork probe assembled structure.

**Figure 12:**
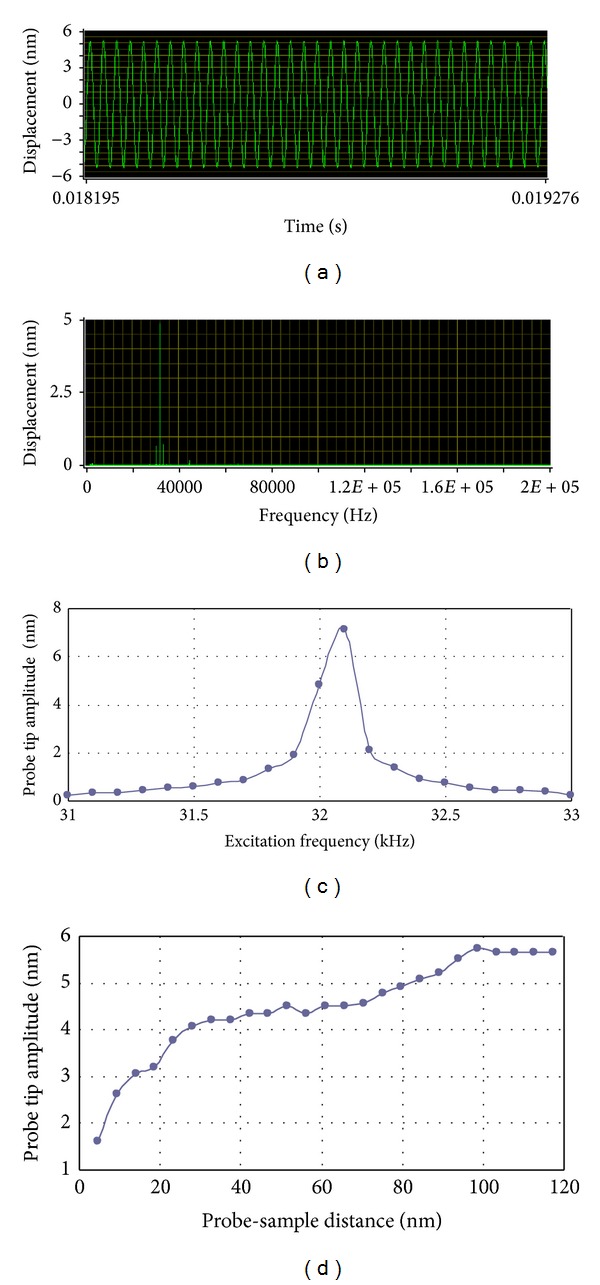
(a) Displacement of the probe tip in a few periods when excited at 32 kHz; (b) spectrum of displacement shown in (a); (c) the frequency-amplitude curve of the TF probe assembled structure in the ambient environment; (d) the amplitude of the probe tip approaching a wet sample surface.

Scanning near-field optical microscopy is a powerful technique having the capability of breaking the diffraction limits by using evanescent waves [76–79]. This is done by conducting a laser beam through a subwavelength fiber probe aperture which is placed in the near-field of the sample surface or conducting a laser beam to illuminate an aperture less probe tip located very close to the sample surface. In a SNOM system, the control of the probe tip at a constant distance away from the sample surface is a critical issue for gaining reliable optical signals. A commonly adopted method uses a tuning fork (TF) driven at its resonance, a probe attached to the one prong of the TF, and a lock-in detection synchronized with the excitation frequency to keep the probe tip scanning at a stable height [80, 81]. In our recent work [82], a single-point LDV is introduced to investigate the dynamic mechanical properties of the TF probe assembled structure where the amplitude and the velocity of the probe were measured in real time.  Figure 11 is a schematic diagram of our setup. The probe is placed at the focal point of the laser beam. The diameter of the focus spots is small enough (8 *μ*m) compared to the dimension of the probe (100 *μ*m) and therefore ensures the signal strength reflected to the sensing head.  Figure 12(a) shows the displacement of the probe tip when excited at 32 kHz and Figure 12(b) shows its frequency spectrum.  Figure 12(c) is the frequency-amplitude curve of the probe tip while the TF probe assembled structure is working under sweep operation. The peak at 32.1 kHz is clear which indicates one of the resonance frequencies of the system.  Figure 12(d) is the displacement curve while the probe tip approaches a wet surface. When the probe tip touches the water film, the vibration amplitude reduces dramatically.

#### 3.3.2. Multipoint LDV for Transient Events Measurement

**Figure 13:**
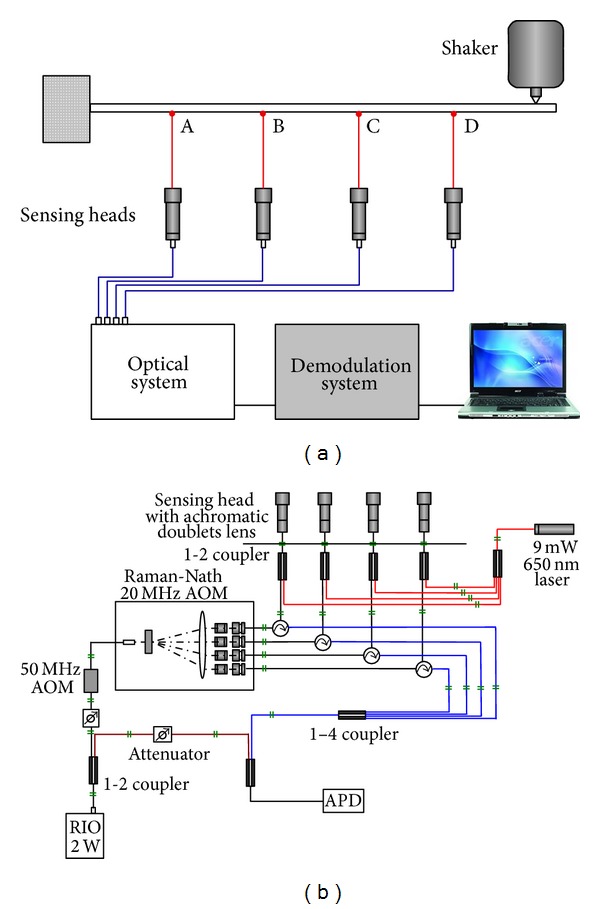
Schematic layout of (a) 4-point laser Doppler vibrometer and (b) its optical system.

Based on the spatially encoded technique, a fiber-based prototype of 4-point self-synchronized LDV was developed. The measurement points can be on different surfaces and/or in arbitrary positions. However, the beam array generated by the AOMs is a regular 1D or 2D pattern and this limits the flexibility of the measurement. In this case we can improve the flexibility by using a fiber-based configuration where different frequency shifts are coupled into the fiber. The reflected object beams are combined with one reference beam. Figures 13(a) and 13(b) show the schematic layout of a 4-point LDV system and its optical design. Four sensing heads are connected with the main optical system and focus the laser beams on different points of the object (points A, B, C, and D). A 4-channel demodulation system was connected to the optical system for real-time decoding.

**Figure 14:**
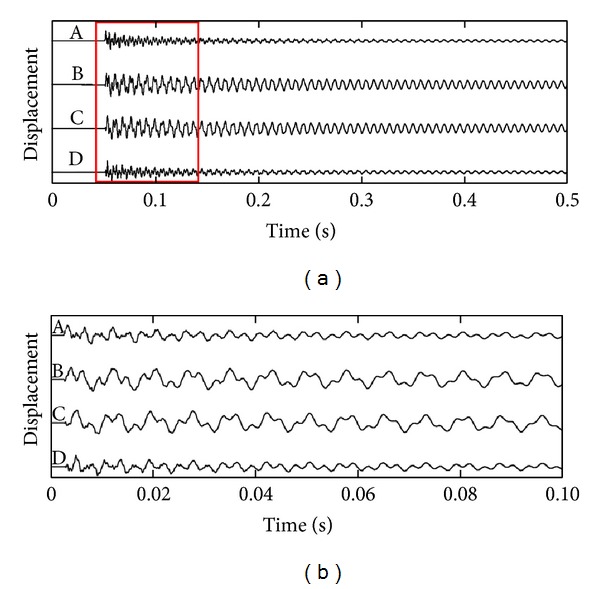
(a) Displacement of points A, B, C, and D in the first 0.5 sec after trigger when the excitation is at point E; (b) the displacement of 4 points in period shown in (a).

**Figure 15:**
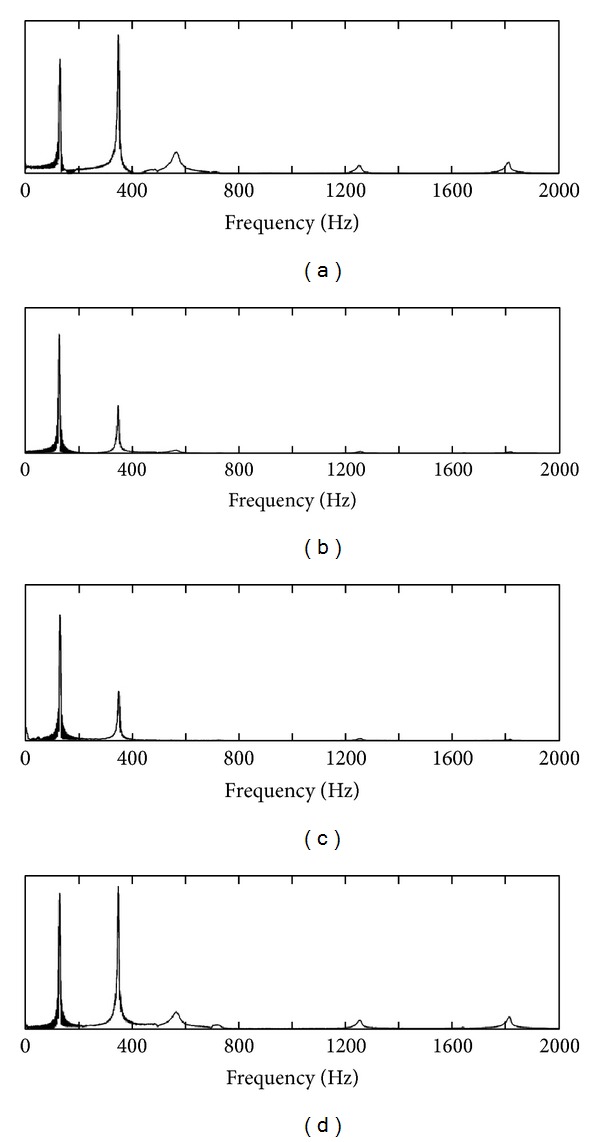
Spectrum displacement shown in Figure 14 on points (a) A, (b) B, (c) C, and (d) D.

Figure 14(a) shows the displacement of points A, B, C, and D in the first 0.5 sec after trigger when pendulum hits point E. The absolute values of the displacements are not indicated as the values vary with different hitting forces.  Figure 14(b) shows the displacement in the period as indicated in Figure 14(a). The displacements of these four points contain different frequencies.  Figure 15 shows the spectrum of the displacement on four measurement points. The spectra are calculated in the range of 0 to 2000 Hz. On points A and D, five peaks at 131.5 Hz, 351.2 Hz, 565.9 Hz, 1253 Hz, and 1813 Hz can be clearly observed, which indicate the first five resonance frequencies of the structure. On points B and C, only two high peaks at 131.5 Hz and 351.2 Hz are observed.

It is worth noting that only one photodetector is used in the prototype and thus synchronization is never an issue; we call it self-synchronizing. This configuration is very useful when the transient response of different points is measured, especially when the propagation of the wave in a structure is studied.

## 4. Conclusions

Optical dynamic measurement based on interferometry can be classified into camera-based and detector-based techniques. The bottleneck of camera-based methods is in the temporal domain, which is mainly limited by the frame rate of the camera. On the other hand, the limitation of the detector-based techniques is in spatial domain. This paper reviewed the main developments of these two methods in the last ten years. With the current status of the hardware, several technologies were introduced to increase the measurement range and seek the balanced resolution along spatial or temporal axis. The main purpose of the research is to reduce the huge gap in the spatiotemporal domain between these two techniques as shown in Figure 1, so that they can meet the requirements of industrial applications.

## Figures and Tables

**Table 1:** Comparison of spatial-carrier and temporal-carrier-based techniques in interferometry.

Factors	Spatial-carrier-based technology	Temporal-carrier-based technology
Processing domain	*x- *and *y-*axis	*t-*axis
Dimension	2-D	1-D
Reflectivity	Affected by nonuniform reflectivity	Not affected
Height step	Affected by height step of test objects	Not affected
Shape	Affected by irregular shape of surface	Not affected
Retrieved phase map quality	Poor and affected by speckle noise when laser is used	Much better than spatial carrier as the process is 1-D along time axis.
Measurement range in temporal domain	Determined by Nyquist sampling theorem	Affected by temporal carrier and much less measurement range than spatial carrier
